# Risk factors associated with a high incidence of sexually transmitted infections in Beitbridge, Zimbabwe

**DOI:** 10.4102/curationis.v45i1.2191

**Published:** 2022-07-28

**Authors:** Anelle Siziba, Wilfred N. Nunu, Nicholas Mudonhi, Vuyelwa Ndlovu, Ofhani Munyai, Brighton Ndlovu, Edmond Sanganyado

**Affiliations:** 1 Department of Environmental Science and Health, Faculty of Applied Sciences, National University of Science and Technology, Bulawayo, Zimbabwe; 2 Scientific Agriculture and Environment Development Institute, Bulawayo, Zimbabwe; 3 Department of Public Health, Municipality of Beitbridge, Beitbridge, Zimbabwe; 4 Department of Applied Mathematics and Statistics, Midlands State University, Gweru, Zimbabwe; 5 Guangdong Provincial Laboratory of Marine Biotechnology, Institute of Marine Science, Shantou University, Guangdong, China

**Keywords:** sexually transmitted infections (STIs), sexual behaviour, sex workers, high STI incidence, Beitbridge, Zimbabwe

## Abstract

**Background:**

Sexually transmitted infections (STIs) are a major public health challenge, particularly in developing countries where the health infrastructure is often poor. Despite having a number of interventions in Beitbridge (such as the 24-h wellness centre), Zimbabwe, the incidence and prevalence of STIs is increasing.

**Objectives:**

This study, therefore, aims to assess the risk factors associated with the high incidence of STIs in urban Beitbridge.

**Method:**

A case-control study was conducted on 30 respondents who had suffered from STIs (selected from the registers at Beitbridge hospital and followed up) and 90 respondents who had not suffered from STIs (from the community) who resided in Beitbridge for at least 6 months and this excluded all those who were in transit. The factors contributing to the high incidence of STIs were identified using a pretested interviewer-administered semi-structured questionnaire in conjunction with a Likert scale to establish the attitudes and risk behaviours of the respondents on STIs.

**Results:**

A significant association was observed between high STI incidences and the nature of occupation (odds ration [OR]: 3.8), area of residence (OR: 2.78), number of sexual partners (OR: 7.56), payment demanded for sex (OR 22), condom use (OR: 3.7), alcohol use (OR: 2.86), and suspicion that partners had other sexual companions (OR: 15.5). Furthermore, a larger proportion of controls were knowledgeable on STIs as compared to the cases who participated in the study.

**Conclusion:**

There is a need to develop awareness strategies that foster safe sexual practices, particularly among populations who do not choose abstinence or a single sexual partner lifestyle.

## Introduction

Sexually transmitted infections (STIs) are a major public health challenge, particularly in developing countries where the health infrastructure is often poor (Marshall et al. 2009). Generally it is reported that at least one million people are infected with curable STIs each day worldwide of which at least 10% of these cases are from sub-Saharan Africa (Newman et al. 2015; Seidu et al. 2020; Torrone et al. 2018; World Health Organization 2016). However, when left untreated, STIs may lead to acute diseases such as cervical cancer and pelvic inflammatory disease, infertility, and sometimes death (Jung 2019; Naidoo et al. 2014). Sexually transmitted infections may cause adverse birth outcomes and mother-to-child transmission as well as other severe reproductive health issues in pregnant women (Kurewa et al. 2010). Furthermore, STIs have been shown to increase the transmission and acquisition of the human immunodeficiency virus (HIV) (Reynolds et al. 2006).

The incidence of STIs is often influenced by structural factors such as economic, political, social, and organisational environments that define the context of the STI risk in the population (Marshall et al. 2009). For example, several studies have shown that poor health infrastructure, high cost of healthcare, lack of STI surveillance systems, poor sexual health knowledge and practices, and migratory behaviours are associated with high STI incidences in sub-Saharan Africa (Chirwa 2020; Kurewa et al. 2010; Mohammed et al. 2019; Nunu et al. 2020; Olusola et al. 2019). Previous studies have found that sex workers and truck drivers were the high-risk groups for STIs as they often have multiple sex partners and are unprotected (Botão et al. 2016; Makhakhe et al. 2017). Urban centres along trade routes are important hotspots of high STI incidence because sex workers are often concentrated along trade routes used by truck drivers (Delany-Moretlwe et al. 2014; Gysels, Pool & Bwanika 2001). However, there are few studies that document the risk factors associated with high STI incidences in port of entries and exits between countries.

**FIGURE 1:**
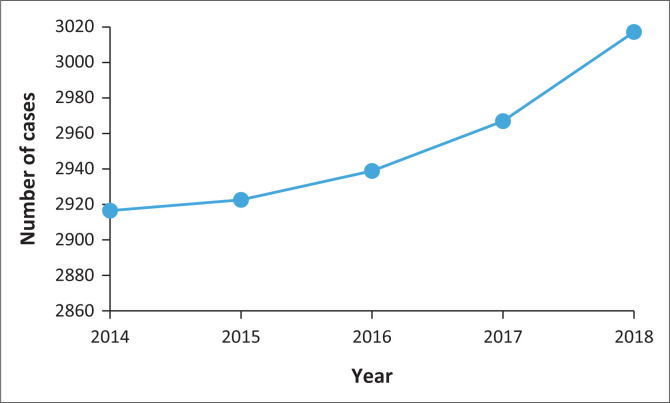
Incidences of sexually transmitted infections in Beitbridge, Zimbabwe from 2014 to 2018.

Beitbridge town in Zimbabwe is an important trade route connecting South Africa to several countries north of Limpopo such as Zimbabwe, Zambia, and Malawi. Previous studies have shown that increase in knowledge and access to sexual health centres were effective in reducing STI incidences (Boyce, Katz & Standley 2019; Bunnell et al. 1999; Chirwa 2020). Interestingly, the STI incidences increased in Beitbridge despite the surge in collaborative STI prevention programmes spearheaded by non-governmental organisations, the local government, and the Ministry of Health and Child Welfare (Figure 1). For example, since 2016, several 24-h wellness centres for STI treatment and testing were established around the town while STI awareness campaigns were intensified. The aim of this study was to assess the risk factors associated with the high STI incidence in Beitbridge, Zimbabwe.

## Methods

### Setting

Beitbridge district is in the southern region of Zimbabwe and is divided into 15 administrative districts, with the Beitbridge town located in Ward 4. It serves as an important trade route for most countries in the Southern African Development Community trade block as it is at the border between South Africa and Zimbabwe. Beitbridge is one of the busiest border posts in the region serving 170 000 travellers, 2100 buses, 2000 private cars, and 15 000 trucks each month. Hence, the freight industry is one of the most important sectors in the town. As a result, sex trade is high in the town because of poverty and poor economic opportunities for women.

### Study design

A case-control study was conducted to identify the STI incidence risk factors. The case-control approach helped in the identification and quantification of the risk factors associated with high STI incidences through systematic comparisons between cases and controls (Schulz & Grimes 2002). This study design is also affordable as it did not require conducting follow-ups on respondents which can be a major hindrance in resource-strapped communities like Beitbridge.

### Study population and sampling

In 2012, Beitbridge had a population of more than 42 000 and an estimated 17 000 people are in transit between Zimbabwe and South Africa daily. However, this study targeted those participants (cases and controls) who had resided in Beitbridge for at least 6 months by the time the study was conducted. Those who were in transit and those who had resided for less than 6 months were excluded from the study. This was because the study sought to explore the risk factors within that town and the best people to answer the research questions about different aspects of activities in that town are those who would have resided there for a significant time frame. The target minimum sample size (to make meaningful inferences) of 120 was determined using the R38 sample size calculator (EPI Info^TM^ software, U.S. Department of Health & Human Services, United States [US]) at 95% confidence interval (CI), 9% width of confidence, and the expected value of 50%. A case was defined as a person aged 16–50 years treated for an STI at the Beitbridge District Hospital between January and December 2018. The cases were then matched to controls in the same age range, who were defined as people who stayed in Dulibadzimu, Beitbridge. The cases were corresponded to controls at a ratio of 1:3 giving a target of 30 cases and 90 controls. Only the cases that were recorded in the registers that were kept at Beitbridge Hospital were deemed eligible to participate in the study. The 30 cases were recruited from the registers kept at the Beitbridge District Hospital using random numbers. Potential respondents were followed-up to their place of residence. Only the respondents who gave the consent and had stayed in Beitbridge for at least 6 months were included in the study.

### Data collection and analysis

Data was collected from the respondents using a piloted researcher administered questionnaire adapted from the Centre for Disease Control and Prevention. The questionnaire consisted of three sections, namely Section A (demographic and socio-economic characteristics), Section B (knowledge about STIs), and Section C (health-seeking and risky sexual behaviour). The questionnaire had a total of 30 questions and took an average 15–25 min to administer. The questionnaire was translated into isiNdebele and ChiShona, which are the major dialects that are spoken in the country and Beitbridge.

The respondents were asked a total of 18 knowledge questions that were scored based on predetermined answers with participants choosing what they felt best corresponded with their beliefs and behaviours. If a person answers the question correctly in terms of the current knowledge regarding transmission, acquisition, and prevention of STIs, they were awarded one point and if they answered it wrongly, they were given a zero. After completion of all the questions, the number of correctly answered questions was populated. A Likert scale was then developed as adapted from other studies (Albaum 1997; Nunu, Kativhu & Moyo 2018; Nunu & Munyewende 2017). If one scored between 0–6, they were considered not knowledgeable, 7–9 intermediate, and > 10 knowledgeable. Multiple logistic regression (MLR) analysis was conducted to determine the risk factors that contributed to the high incidence of STIs in Beitbridge. Data was analysed using STATA version 13.

### Ethical considerations

Written permission to carry out the study was sought from the Ministry of Health and Child Care, Beitbridge Town Council and the National University of Science and Technology. Written consent was also sought from the participants themselves and they were made aware of their rights to withdraw at any point when they felt so. The Helsinki declaration on principles to be observed when conducting studies that deals with human subjects was observed throughout the study.

## Results

### Socio-economic characteristics associated with high sexually transmitted infection incidences

Structured questionnaires were used to identify the socio-economic risk factors associated with high incidences of STIs in Beitbridge town. Nature of employment, residence area, affiliation to African Indigenous Churches (African Apostolics), and living with family showed significant association with high STI incidences in Beitbridge (Table 1). The cases were 3.8 and 2.78 times likely to lack formal employment or reside in the high-density suburb of Dulibadzimu compared to the controls, respectively. Interestingly, the cases were 2.36 and 2.06 times, respectively more likely to be professed members of other denominations rather than African Indigenous Churches and that they stayed alone. These results showed that economic, religious, and social activities influence the prevalence and incidence of STIs.

**TABLE 1:** Socio-economic demographic characteristics of cases and controls.

Variable	Cases		Controls		MLR-OR	95% CI
	*n*	%	*n*	%		
**Sex**						
Males	12	40	38	42	-	-
Females	18	60	52	58	1.04	0.45–2.4
**Age**						
16–30	15	50	49	54	1.33	0.56–3.1
31–50	15	50	41	46	-	-
**Marital status**						
Married	16	53	47	52	-	-
Other	14	47	43	48	1.05	0.45–2.34
**Religion**						
African Apostolic	14	47	34	38	-	-
Other denominations	16	53	56	62	2.36	0.5–11.1
**Level of education**						
Never been to school	3	10	6	7	-	-
Primary or better	27	90	84	93	0.326	0.02–5.37
**Occupation**						
Employed	10	34	31	34	-	-
Other	20	66	59	66	3.8	1.56–9.1
**Migrant**					-	-
1–5	12	40	34	38	-	-
>5 years	18	60	56	62	1.09	0.47–2.5
**Who you stay with**						
Family	14	47	61	68	-	-
By myself	16	63	29	32	2.06	0.88–4.8
**Area of residence**						
Dulibadzimu	21	70	78	87	2.78	1.04–7.5
Other residential areas	9	30	12	13	-	-

MLR-OR, multiple logistic regression-odds ratio; CI, confidence interval.

### Sexually transmitted infection knowledge levels

**FIGURE 2:**
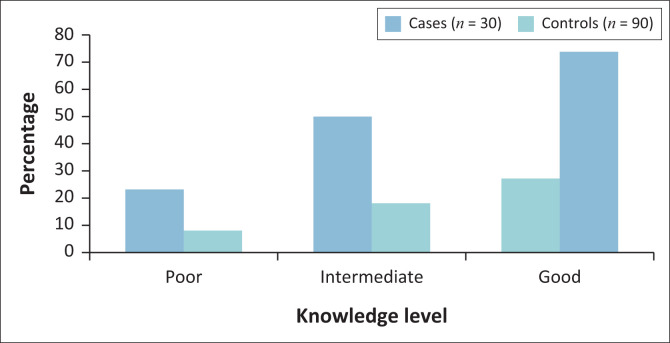
Level of knowledge on sexually transmitted infections of the respondents.

The effect of awareness of STI transmission, treatment, and prevention was assessed using a Likert scale (Figure 2). A large proportion (*n* = 67; 74%) of the controls were proved to be more knowledgeable compared to the proportion (*n* = 8; 27%) of the cases. Furthermore, a smaller percentage of the controls (*n* = 7; 8%) were not knowledgeable compared to the cases (*n* = 7; 23%). These results suggest that knowledge of STI could provide protection from STIs.

### Sexual behaviours

The factors identified to be the major contributors to the high STI incidence were the number of sexual partners, sex trade, condom usage during sexual intercourse, and excessive alcohol consumption (Table 2). The cases were 7.56 times more likely to have had at least two sexual partners compared to the controls. Furthermore, the cases were 22 times more likely to be involved in sexual activities for monetary gains compared to the controls. These results suggest that risky sexual behaviours were indicators of high STI incidences in Beitbridge.

**TABLE 2:** Risky sexual behaviours of the cases and controls in Beitbridge, Zimbabwe.

Variable	Cases		Controls		MLR-OR	95% CI
	*n*	%	*n*	%		
**Sexual partners**						
1	8	27	66	73	-	-
> 2	22	73	24	27	7.56	2.9–19.2
**Relationship with recent sexual partner**						
Spouse	19	63	70	78	-	-
Friend	11	37	20	22	1.14	0.5–2.6
**Payment demanded for sex**						
Yes	10	33	2	2	22	4.5–108.3
No	20	67	88	98	-	-
**Condom use**						
Yes	4	13	33	37	-	-
No	26	87	57	63	3.7	1.2–11.7
**Alcohol use**						
Yes	13	43	19	21	2.86	1.18–6.9
No	17	57	71	79	-	-
**Regular sexual partner**						
Yes	21	70	46	51	-	-
No	9	30	44	49	2.23	0.92–5.4
**Cohabiting with regular partner**						
Yes	7	33	8	17	-	-
No	14	77	38	83	2.375	0.73–7.8
**Suspects partner is promiscuous**						
Yes	19	63	9	10	-	-
No	11	37	81	90	15.5	5.6–42.7
**Sexual harassment on females**						
Yes	7	39	0	-	-	-
No	11	61	52	100	†	-

MLR-OR, multiple logistic regression-odds ratio; CI, confidence interval.

† , the outcome is equal to that of the comparison group so odds could not be computed.

## Discussion

The study found that the nature of employment was a significant factor that contributed to the high incidence of STIs. Those that were not formally employed had to find other sources of livelihoods of which prostitution was one of them. These findings are supported by Hunter (2007) in a study that was conducted in South Africa which reported that prostitution is one of the sources of livelihoods particularly in unemployed women (Hunter 2007). These findings agree with that of previous studies in Uganda (Gysels et al. 2001), South Africa (Makhakhe et al. 2017; Naidoo et al. 2014), and South Korea (Jung 2019) that found an association between prostitution and poverty. The unemployment rate in Zimbabwe is estimated at above 90% and this results in most people pursuing informal trades including sex work (Mujeyi & Sadomba 2019).

The area of residence was also significantly associated with the high incidence of STIs as the odds were 2.78 times more likely that the cases were residing in Dulibadzimu residential area as compared to other suburbs. Dulibadzimu is the only high-density suburb in Beitbridge. Most people in Beitbridge stay in Dulibadzimu because it has cheaper accommodation. These findings are reinforced by Evans and Saegert (2000) who found that low-income families tend to crowd in high-density suburbs that are affordable.

A larger proportion of the controls were knowledgeable as compared to the cases. It has been reported that knowledge about STIs plays an important role in mitigating its impacts (Hemalatha et al. 2011). Knowledgeable people are bound to take necessary steps in preventing themselves from contracting STIs (Hemalatha et al. 2011). On the other hand, lack of knowledge on STIs places one at a higher risk of contracting STIs (Hemalatha et al. 2011) as evidenced by the higher proportion of cases who were not knowledgeable as compared to the controls in our current study. The cases were 7.56 times more likely to have slept with at least two sexual partners. Having multiple sexual partners increases the risk of contracting STIs (Rosenberg et al. 1999). The more the number of sexual partners the higher the risk of contracting and spreading of STIs (Rosenberg et al. 1999). Different studies have reported that being faithful to one sexual partner reduces the risk of contracting and spreading of STIs (Desiderato & Crawford 1995; Joffe et al. 1992; Setswe 2007).

The cases were 22 times more likely to demand money for sexual activities as compared to the controls. The same cases were 3.7 and 2.86 times more likely not to use a condom and consume alcohol, respectively. Most of the cases were engaged in prostitution which is usually associated with risky sexual behaviours and consumption of alcohol (Cusick 1998; Gossop et al. 1995). Furthermore, our study found that the cases were 15.5 times not to suspect that their partners were promiscuous. This shows that they do not really care what their partners do out there as most have transactional sex and multiple partners (Sato 2016). They only care about whether their needs are taken care of (Sato 2016).

## Conclusion

In conclusion, this study found that the nature of employment, knowledge of STIs, area of residence, multiple sexual partners, transactional sex, failure to use condoms, alcohol consumption and failure to care about sexual partners’ activities were significantly associated with a high incidence of STIs. Therefore, there is a need to conduct intense awareness campaigns regarding the risk of having multiple sex partners, and about alcohol intake management, and safe sex practices such as condom use. It is evident that abstinence is not possible for those who depend on proceeds from sexual activities. Therefore, safe sexual practices should be encouraged.
